# Glial-neuronal signaling mechanisms underlying the neuroinflammatory effects of manganese

**DOI:** 10.1186/s12974-018-1349-4

**Published:** 2018-11-21

**Authors:** Katriana A. Popichak, Maryam F. Afzali, Kelly S. Kirkley, Ronald B. Tjalkens

**Affiliations:** 1 Department of Environmental and Radiological Health Sciences, College of Veterinary Medicine and Biomedical Sciences, Colorado State University, 1680 Campus Delivery, Physiology Building, Room 101, Fort Collins, CO 80523-1680 USA; 2 Department of Microbiology, Immunology and Pathology, College of Veterinary Medicine and Biomedical Sciences, Colorado State University, Fort Collins, CO USA; 3 Department of Biomedical Sciences, College of Veterinary Medicine and Biomedical Sciences, Colorado State University, Fort Collins, CO USA

**Keywords:** Neuroinflammation, Manganism, Astrocyte, Glial-glial communication, Glial-neuronal communication, NF-κB, CCL2

## Abstract

**Background:**

Exposure to increased manganese (Mn) causes inflammation and neuronal injury in the cortex and basal ganglia, resulting in neurological symptoms resembling Parkinson’s disease. The mechanisms underlying neuronal death from exposure to Mn are not well understood but involve inflammatory activation of microglia and astrocytes. Expression of neurotoxic inflammatory genes in glia is highly regulated through the NF-κB pathway, but factors modulating neurotoxic glial-glial and glial-neuronal signaling by Mn are not well understood.

**Methods:**

We examined the role of NF-κB in Mn-induced neurotoxicity by exposing purified microglia, astrocytes (from wild-type and astrocyte-specific IKK knockout mice), and mixed glial cultures to varying Mn concentrations and then treating neurons with the conditioned media (GCM) of each cell type. We hypothesized that mixed glial cultures exposed to Mn (0–100 μM) would enhance glial activation and neuronal death compared to microglia, wild-type astrocytes, or IKK-knockout astrocytes alone or in mixed cultures.

**Results:**

Mixed glial cultures treated with 0–100 μM Mn for 24 h showed the most pronounced effect of increased expression of inflammatory genes including inducible nitric oxide synthase (*Nos2*), *Tnf*, *Ccl5*, *Il6*, *Ccr2*, *Il1b*, and the astrocyte-specific genes, *C3* and *Ccl2*. Gene deletion of IKK2 in astrocytes dramatically reduced cytokine release in Mn-treated mixed glial cultures. Measurement of neuronal viability and apoptosis following exposure to Mn-GCM demonstrated that mixed glial cultures induced greater neuronal death than either cell type alone. Loss of IKK in astrocytes also decreased neuronal death compared to microglia alone, wild-type astrocytes, or mixed glia.

**Conclusions:**

This suggests that astrocytes are a critical mediator of Mn neurotoxicity through enhanced expression of inflammatory cytokines and chemokines, including those most associated with a reactive phenotype such as CCL2 but not C3.

## Background

Manganese (Mn) is an essential trace element primarily acquired through diet. However, increased exposures in juveniles and adults lead to inflammation and neuronal injury in the cortex and basal ganglia that can cause irreversible neurodegeneration associated with cognitive and motor deficits. Sources of excess Mn exposure include soy-based infant formula [1], well water [2], and industrial activities such as mining [3] and welding [4]. Activation of astrocytes and microglia in response to Mn neurotoxicity can lead to overproduction of neurotoxic levels of reactive oxygen and nitrogen species (ROS, RNS) [5], as well as inflammatory cytokines such as TNF [6]. Additionally, astrocytes concentrate Mn through plasma membrane divalent metal transports, which increase oxidative stress and decrease their capacity for neuronal trophic support by affecting key metabolic coupling pathways such as glutamate uptake and glutathione synthesis [7, 8]. The combination of enhanced inflammatory gene expression and decreased trophic support may therefore cause a reactive phenotype that promotes neuronal injury.

A number of studies have demonstrated that Mn-induced glial activation is exacerbated by glial-derived pro-inflammatory factors that damage neurons. Data from our laboratory recently demonstrated that NF-κB signaling in microglia plays an essential role in inflammatory responses in Mn toxicity by regulating cytokines and chemokines that amplify the activation of astrocytes [6]. Although this demonstrates that nuclear factor kappa B (NF-κB) signaling in microglia is essential to inflammatory activation of astrocytes, subsequent effects of astrocytes on microglia and ultimately on neuronal cell death are less well understood. It was reported that Mn exposure induces activation of microglia and signs of dystrophy including increased iron-mediated oxidative stress in the substantia nigra of non-human primates [9], as well as microglia-induced degeneration of dopaminergic neurons in rats [10]. Previous studies from our laboratory and others demonstrated that expression of inducible nitric oxide synthase (iNOS/NOS2) and NO production in astrocytes causes injury to surrounding neurons in Mn-exposed mice [11–14]. NOS2 and many other pro-inflammatory factors are highly regulated in glial cells by NF-κB, consistent with data reporting that the NF-κB-mediated pro-inflammatory cytokines CCL2, CCL5, and TNF released by astrocytes are associated with Mn neurotoxicity in in vitro studies of murine glia [5, 15, 16].

However, only recently has research begun to establish how the communication between both glial cell types mediates Mn-induced neuronal injury [17]. The astrocyte-specific chemokine, CCL2, is regulated by NF-κB and induces microglial activation in a surgery-induced cognitive dysfunction and neuroinflammatory model, suggesting not only that astrocytes and microglia communicate during stress and injury, but also that NF-κB-mediated factors contribute to glial inflammation. NF-κB is activated in glia in response to Mn, oxidative stress, and other neurotoxic exposures [15, 16, 18]. We recently reported that NF-κB activation in microglia amplifies the inflammatory response of astrocytes to Mn toxicity, resulting in overproduction of inflammatory cytokines and chemokines such as TNF, IL1, and IL6 [6]. However, it remains to be determined which NF-κB-regulated inflammatory signaling molecules in astrocytes modulate the inflammatory response of microglia, as well as the effect of this signaling on neuronal injury.

We therefore examined the role of NF-κB in Mn-induced neurotoxicity by exposing pure microglia, astrocytes (from wild-type and astrocyte-specific IKK/NF-κB knockout mice), and mixed glial cultures to varying concentrations of Mn and then treating neurons with the resultant glial-conditioned media (GCM) of each cell type. We hypothesized that Mn would enhance expression of inflammatory cytokines in mixed cultures of astrocytes and microglia to a greater extent than in either cell type alone and would similarly increase neuronal cell death. We measured expression of NF-κB-regulated inflammatory genes by qPCR in glial cells, as well as levels of secreted cytokines in GCM from both WT or IKK KO astrocytes and microglia using spotted array-based ELISA. Mn exposure enhanced expression of multiple inflammatory cytokines and chemokines in mixed glial cultures, which was inhibited by pharmacologic inhibition or genetic inhibition of NF-κB. Additionally, Mn-stimulated GCM increased neuronal apoptosis, which was attenuated by inhibition of NF-κB in astrocytes, suggesting that activation of this signaling pathway in astrocytes is a critical mediator of glial reactivity and neuronal injury from Mn through release of cytokines and chemokines that amplify activation of microglia.

## Materials and methods

### Materials

All general chemical reagents including MnCl_2_ and antibiotics were purchased from Sigma Aldrich (St. Louis, MO). Fluorescent antibodies and dyes were purchased from Life Technologies (Carlsbad, CA). Cell culture medium was acquired from Hyclone (Logan, UT) or Gibco (Life Technologies, Carlsbad, CA). For immunofluorescence studies and flow cytometric experimentation and live-cell imaging of primary neurons, Annexin V-iFluor™ 647 conjugate was purchased from AAT Bioquest (Sunnyvale, CA), and Propidium Iodide and Caspase-3/7 Green were purchased from Life Technologies (Carlsbad, CA).

### Primary glial and neuronal isolation

Cortical glia and neurons were isolated from 1-day-old C57Bl/6 or transgenic mouse pups according to procedures described previously [6, 19], and purity confirmed through immunofluorescence staining using antibodies against GFAP and IBA1 [20]. Mixed glial cultures were also established from astrocyte-specific IKK2 knockout mice, which were generated in our laboratory by crossing mice expressing a *loxP*-targeted (floxed) *Ikk2* allele [21] with mice expressing the human *Gfap* promoter driving expression of cre recombinase [22]. Progeny were bred to homozygosity for the floxed-*Ikk2* allele, and both male and female littermates from the F4 generation were utilized for cell isolations. Briefly, pups were euthanized by decapitation under isofluorane anesthesia, and cortices (astrocytes) were rapidly dissected out, and meninges removed. Tissue was subject to digestion with Dispase (1.5 U/ml), and a complete media change 24 h after plating to remove non-glial cell types from glial cultures and glia from neuronal cultures. Glial cultures were maintained at 37 °C and 5% CO_2_ in minimum essential media supplemented with 10% heat-inactivated fetal bovine serum and a penicillin (0.001 mg/ml), streptomycin (0.002 mg/ml), and neomycin (0.001) antibiotic cocktail (PSN). Neuronal cultures were maintained at 37 °C and 5% CO_2_ in neurobasal media supplemented with HEPES, B27, and PSN. Cell media was changed 24 h prior to all treatments. All animal procedures were approved by the Colorado State University Institutional Animal Care and Use Committee and were conducted in accordance with published NIH guidelines. Neuro-2a cells (N2A) were cultured as previously described [23].

### Treatments and glial-conditioned media experiments

Glia were treated with MnCl_2_ (solubilized in saline) (0–100 μM) for 8 h similarly to methods and experimentation previously described [6]. In brief, prior to mRNA assessment or conditioned media experiments, glia were seeded onto six-well tissue culture plates at approximately 3 × 10^5^ cells per well, grown to confluence and treated with saline or 100 μM MnCl_2_ (in 2 ml total of cell culture medium adequate for growth/health of glia and survival/health of neurons; see the “Materials and methods” section) for 8 h. To inhibit NF-κB signaling in glia, cells seeded in six-well plates were treated with 5 μM Bay 11-7082 (Bay11; Sigma) or the vehicle dimethyl sulfoxide (DMSO; Sigma) at 0.05% in complete media for 3 h. Exposure to Bay11 or DMSO for 3 h had no effect on cell viability. Media was removed prior to 8 h treatment with Saline or MnCl_2_. Conditioned media (denoted GCM for mixed glia-conditioned media, ACM for astrocyte-conditioned media, and MCM for microglia-conditioned media) were pooled per treatment and centrifuged at 800×*g* for 10 min to remove detached cells. N2As were seeded in six-well tissue culture plates at 1 × 10^5^ cells per well for flow cytometry experiments or 5 × 10^3^ cells per well of 96-well tissue culture plate for Presto Blue Viability Assay 24 h prior to 48 h exposure to conditioned media. Primary neurons were seeded in four-well chamber slides for live-cell imaging at 1 × 10^5^cells per well and 2 × 10^5^cells per well of a 96-well tissue culture plate for Presto Blue Viability Assay 10 days prior to 48 h exposure to conditioned medium. N2As and primary neurons were then assessed for viability and cell death markers.

### Real-time RT-PCR and qPCR array analysis

Confluent mixed glia, purified astrocytes, or purified microglia were treated with MnCl_2_ (0–100 μM) for 8 h prior to RNA isolation. RNA was isolated using the RNEasy Mini kit (Qiagen, Valencia, CA), and purity and concentration were determined using a Nanodrop ND-1000 spectrophotometer (NanoDrop Technologies, Wilmington, DE). Following purification, RNA (250–1000 ng) was used as template for reverse transcriptase (RT) reactions using the iScript RT kit (BioRad, Hercules CA). The resulting cDNA was immediately profiled for mRNA expression according to the 2-ΔΔCT method [24]. Primer sequences for all genes profiled are provided in Table 1.

**Table 1:** Primer table. Primer sequences of measured genes in qPCR experiments

Gene	Accession no.	Primer sequence (5′–3′)	Length (bp)
*Nos2*	NM_010927.3	For: TCA CGC TTG GGT CTT GTT	149
		Rev: CAG GTC ACT TTG GTA GGA TTT	
*Tnfα*	NM_013693.3	For: CTT GCC TGA TTC TTG CTT CTG	140
		Rev: GCC ACC ACT TGC TCC TAC	
*Il1-β*	NM_008361.3	For: GCA GCA GCA CAT CAA CAA G	90
		Rev: CAC GGG AAA GAC ACA GGT AG	
*Ccl2*	NM_011331.2	For: TTAAAAACCTGGATCGGAACCAA	121
		Rev: GCATTAGCTTCAGATTTACGGGT	
*Ccl5*	NM_013653.3	For: GCT GCT TTG CCT ACC TCT CC	104
		Rev: TCG AGT GAC AAA CAC GAC TGC	
*Il-6*	NM_031168.1	For:CTG CAA GAG ACT TCC ATC CAG	131
		Rev:AGT GGT ATA GAC AGG TCT GTT GG	
*β-actin*	NM_007393.3	For: GCT GTG CTA TGT TGC TCT AG	117
		Rev: CGC TCG TTG CCA ATA GTG	
*Hprt*	NM_013556.2	For: TCA GTC AAC GGG GGA CAT AAA	142
		Rev: GGG GCT GTA CTG CTT AAC CAG	
*C3*	NM_009778.3	For: GAG CGA AGA GAC CAT CGT ACT	83
		Rev: TCT TTA GGA AGT CTT GCA CAG TG	
*Ccr2*	XM_011243064.2	For: ATC CAC GGC ATA CTA TCA ACA TC	89
		Rev: TCG TAG TCA TAC GGT GTG GTG	

List of qPCR primer sequences

### Presto Blue viability assay

N2A cells (5000 cells per well) or primary neurons (2 × 10^5^cells/well) were grown or plated on 96-well plates for 24 h or 10 days, respectively, before treatment with GCM. After 48 h, cells were imaged using the PrestoBlue Cell viability reagent (Life Technologies, Carlsbad, CA) per the manufacturer’s protocol.

### Spotted protein array ELISA assays

Measurement of cytokines in glia-conditioned media (resultant from treatments for mRNA expression profiling of glia) was sampled from glia prior to application on neurons and stored at − 80 °C. Stored media was thawed, and cytokines were measured using a custom mouse 7-plex ELISA (Q-Plex™ Mouse Cytokine Arrays, Quansys Biosciences, Logan, UT) according to manufacturer instructions and imaged on a ChemiDoc XRS (Life Science Research, Hercules, CA) to capture images. Levels of cytokines and chemokines were calculated from standard curves using Q-View imaging software (Quansys Biosciences, Logan, UT).

### RNA interference

RNA interference (siRNA, small interfering RNA) oligonucleotides were purchased from Integrated DNA Technologies (IDT DNA, Coralville, IA). RNAi duplexes were designed against splice common variants of the target gene and were validated using a dose-response assay with increasing concentrations of the suspended oligo (900–1200 ng/ml) using a standard scrambled dicer substrate RNA (DsiRNA) as control. RNAi oligonucleotides were transfected using the TransIT-X2 delivery system (Mirus Bio, Madison, WI) 48 h before 100 μM MnCl_2_ treatment. Separate siRNA systems were used to ensure specific knockdown of *Ccl2* and *C3* mRNA. The Ccl2 dsiRNA duplex sequences are (5′➔3′) UGAAGCUAAUGCAUCCACUACCUTT; UAAACAAUACCUUGGAAUCUCAAACAC (IDT DsiRNA; denoted siCcl2). The C3 dsiRNA duplex sequences are (5′➔3′) UAAUAAAGCUUCAGUUGUAUUUCAA; UUGAAAUACAACUGAAGCUUUAUUAGA (IDT DsiRNA; denoted siC3).

### Flow cytometry

The percent of Annexin-V positive (+) and Propidium Iodide positive (+) in neuroblastoma (N2A) cultures before and after treatment with or without conditioned media or MnCl_2_ for 48 h followed by flow cytometric analysis as described [6]. Briefly, cells were labeled using Annexin-V and Propidium Iodide (PI) at room temperature for 1 h. After labeling, the cells were washed twice in incubation buffer and resuspended at a final volume of 500 μL of PBS and stored at 37 °C until analysis. Flow cytometry was performed on a Beckman Coulter CyAn ADP flow cytometer operated with Summit Software for data collection at Colorado State University’s Flow Cytometry Core Facility. All further data analysis was done utilizing FlowJo software (version 10.1; FlowJo, Ashland, OR).

### Statistical analysis

Experiments were performed in triplicate, with replicates consisting of independent cultures using a minimum of (*n* = 4) four plates or cover slips per replicate study. Comparison of two means was performed by Student’s *t* test, while comparison of three or more means was performed using one-way ANOVA while those consisting of comparison of three or more among the genetic variations of wild-type and knock-out treatment groups followed the Tukey-Kramer multiple comparison post hoc test using Prism software (v6.0 h, Graphpad Software, Inc., San Diego, CA). For all experiments, data was reported as standard error mean (± S.E.M) and *P* < 0.05 was considered significant, although the level of significance was often much greater (*P < 0.05; **P < 0.01; ***P < 0.001; ****P < 0.0001).

## Results

### MnCl_2_ exposure induces increased inflammatory gene expression in mixed glial cultures

**Fig. 1:**
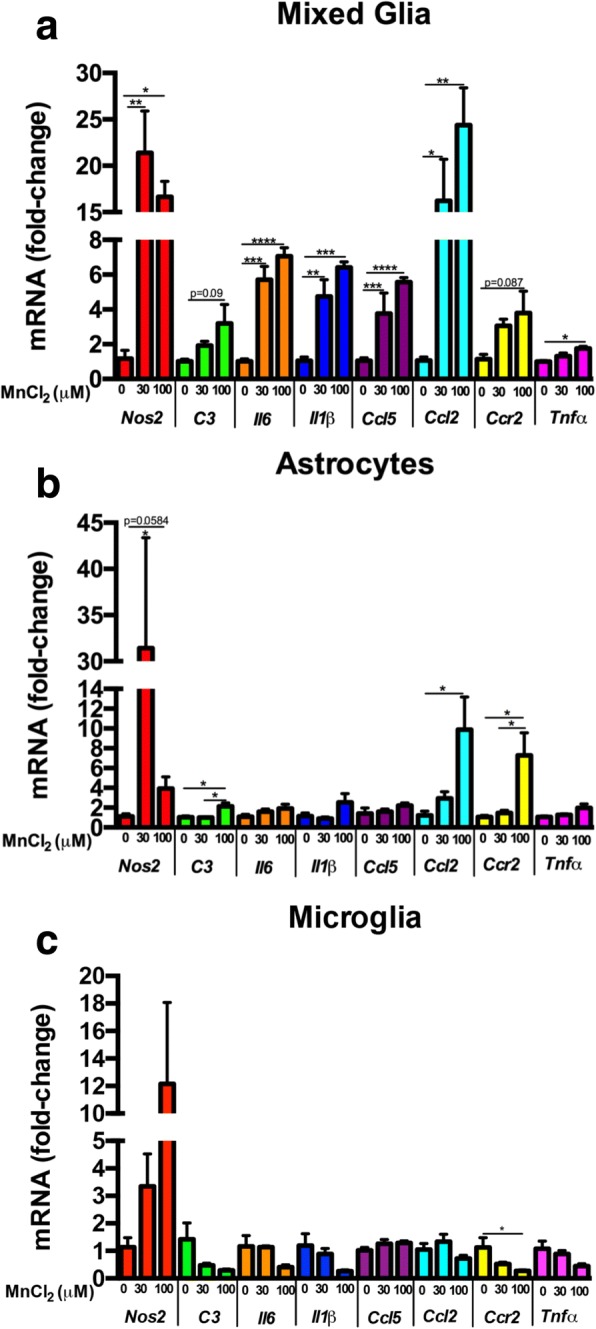
Manganese-induced expression of neuroinflammatory genes in mixed glial cultures and in purified cultures of astrocytes and microglia. Inflammatory gene expression exhibited an overall dose-dependent increase in **a** mixed glia and **b** pure astrocytes and both dose-dependent increases in some genes and decreases in astrocyte-specific *C3*, *Ccl2*, and *Ccr2* in **c** pure microglia. One-way ANOVA analyses performed. Data depicted as ± S.E.M. *P< 0.05; **P < 0.01; ***P < 0.001; ****P < 0.0001 (*n* ≥ 4 per treatment group; across ≥ 3 independent experiments)

To assess inflammatory gene expression following exposure to MnCl_2_, mixed primary glial cultures containing astrocytes and microglia, pure astrocytes, or pure microglia were treated for 24 h prior to profiling expression of inflammatory genes by qPCR. Mn exposure enhanced expression of inducible nitric oxide synthase (*Nos2*) and multiple inflammatory cytokines and chemokines, including the astrocyte-specific inflammatory complement factor, *C3*, as well as *Tnf*, *Ccl2*, and its receptor *Ccr2*, *Ccl5*, *Il6*, and *Il1β*. These genes showed dose-dependent expression in mixed glia, with maximal gene expression at 100 μM MnCl_2_ (Fig. 1a). Pure astrocyte cultures (Fig. 1b) showed less increase in gene expression compared to mixed glia. Purified cultures of microglia (Fig. 1c) displayed less gene expression compared to either pure astrocytes or mixed glial cultures.

### Glia-conditioned media from mixed glial cultures containing both microglia and astrocytes causes more severe neuronal cell death than astrocyte-conditioned media or microglia-conditioned media

**Fig. 2:**
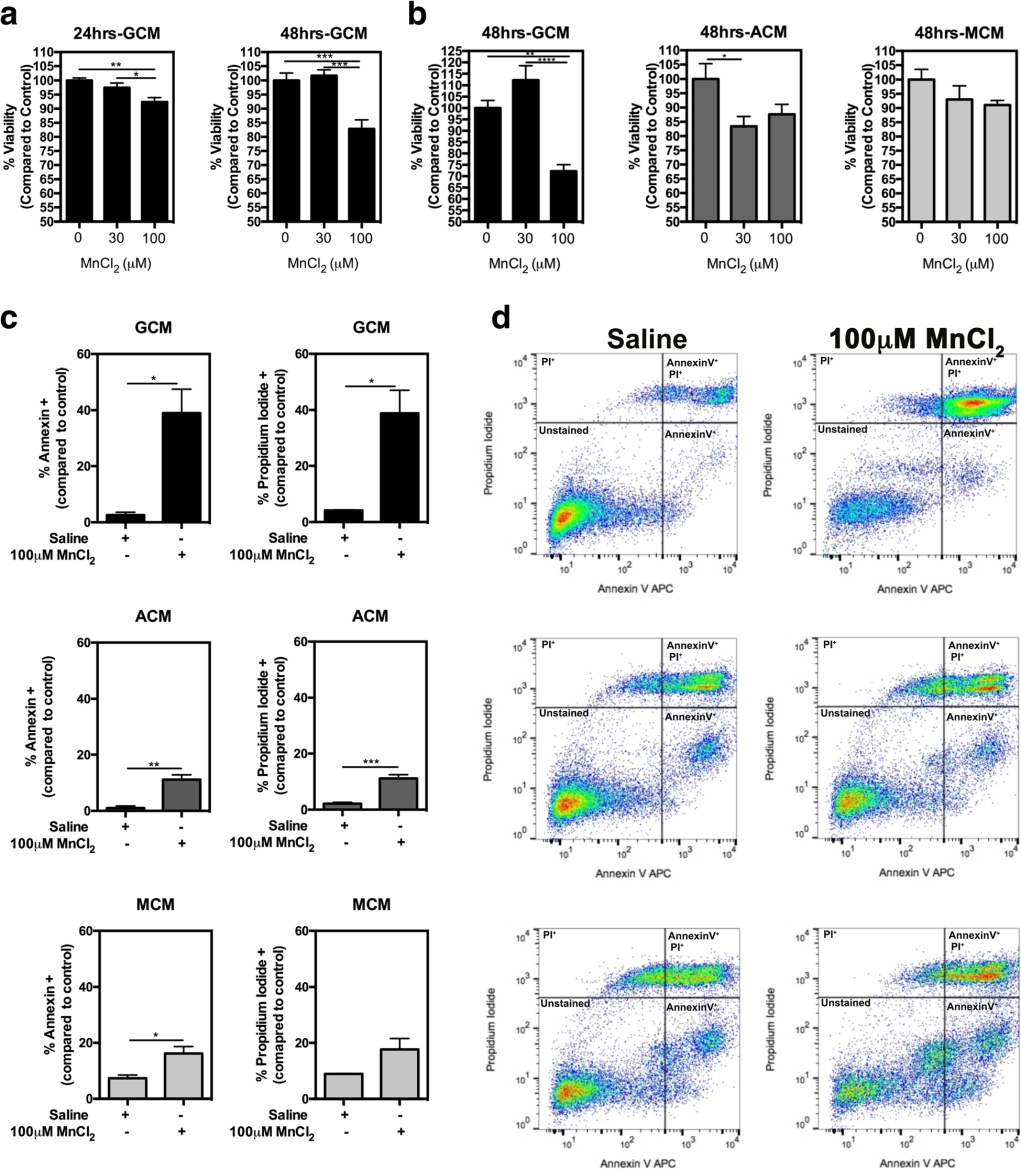
Conditioned media from Mn-treated glial cultures induces cell death in N2A neuronal cells. **a** N2A cells exposed to Mn-exposed GCM for 24 or 48 h showed a dose-dependent decrease in viability at 100 μM MnCl_2_, more so at 48 h. **b** 48-h Mn-treated GCM caused a greater decrease in neuronal viability compared to either ACM or MCM. **c**, **d** (top) Flow cytometry analysis demonstrated that 48-h exposure to 100 μM MnCl_2_-GCM increased apoptosis in N2A cells to a greater extent than ACM (**c**, **d** middle) or **(c** MCM, **d** bottom), based on the number of Annexin V+ and Propidium iodide+ cells. One-way ANOVA analyses performed for experiments comparing three or more treatment groups and *t* test in those comparing two treatment groups. Data depicted as ± S.E.M. *P < 0.05; **P < 0.01; ***P < 0.001; ****P < 0.0001 (*n* ≥ 4 per treatment group; across ≥ 3 independent experiments)

To determine whether MnCl_2_ exposure causes the release of soluble neurotoxic factors from glia, cultures of mixed glia, pure astrocytes, or pure microglia were treated with 0–100 μM MnCl_2_ for 24 h and the resultant glia-conditioned media (GCM) added to cultured neurons (Fig. 2). Conditioned media from mixed glia (GCM) treated with 100 μM significantly decreased neuronal viability following 24 h incubation in culture and was further decreased at 48 h for the 100 μM treatment group (Fig. 2a, left panel). Neuronal cell death following 48 h exposure to 100 μM MnCl_2_-treated conditioned media was greater for GCM (~ 30% decreased viability) than for either astrocyte-conditioned media (ACM) (Fig. 2b, left panel; ~ 15% decreased viability) or microglia-conditioned media (MCM), which did not induce significant cell death despite a trend toward decreasing viability. Flow cytometric analyses of neuronal cells treated for 48 h with GCM-100 μM MnCl_2_ indicated an increase in apoptotic neurons, based on staining for Annexin V and Propidium Iodide (PI), as indicated by quantification bar graphs and depicted in representative histograms. GCM-100 μM MnCl_2_ resulted in 38.98% Annexin-positive and 38.81% PI-positive neurons (Fig. 2c, top panel), as depicted in representative histograms (Fig. 2d, top panel) compared to AMC-100 μM MnCl_2_ which resulted in 11.11% Annexin-positive and 11.13% PI-positive neurons (Fig. 2c, d, middle panel), while MCM-100 μM MnCl_2_ resulted in 16.2% Annexin-positive and 17.66% PI-positive neurons (Fig. 2c, d, bottom panel).

### Pharmacologic inhibition of NF-kB decreases inflammatory gene expression in mixed glia and in purified astrocytes following exposure to MnCl_2_

**Fig. 3:**
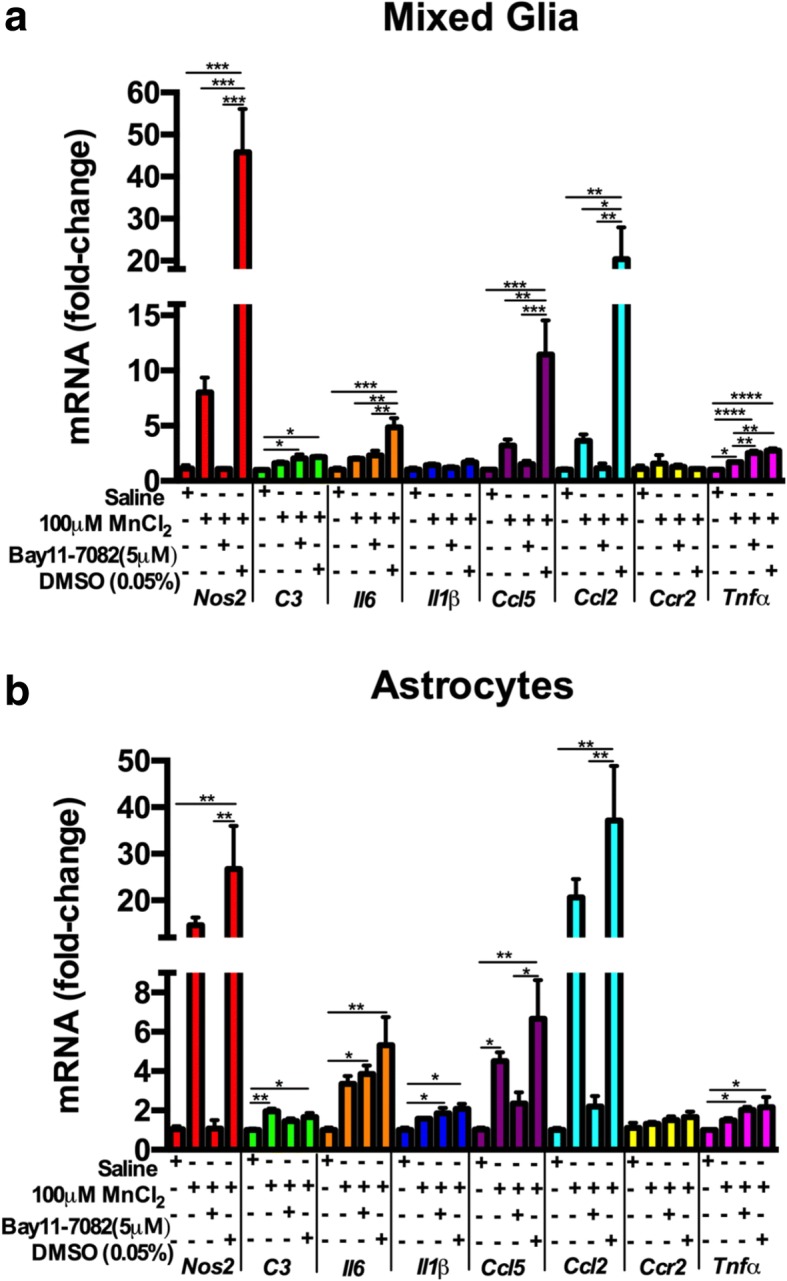
Pharmacologic inhibition of NF-kB decreases inflammatory gene expression upon exposure to 100 μM MnCl_2_. **a** 1 h pretreatment with the NF-κB inhibitor, Bay 11-7082 (Bay-11) [(E)- 3-(4-methylphenyl) sulfonylprop-2-enenitrile], suppressed expression of inflammatory genes in mixed glia following exposure to 100 μM MnCl_2_. **b** MnCl_2_ exposure in pure astrocytes was less than in mixed glia, as were the inhibitory effects of Bay-11, while the DMSO-vehicle control did not suppress inflammatory gene expression in either cell type. One-way ANOVA analyses performed. Data depicted as ± S.E.M. *P < 0.05; **P < 0.01; ***P < 0.001; ****P < 0.0001 (*n* ≥ 4 per treatment group; across ≥ 3 independent experiments)

To determine the function of NF-κB in MnCl_2_-induced inflammatory gene expression in mixed glia or pure astrocytes, we pretreated cell cultures with the NF-κB inhibitor, Bay 11-7082 (Bay-11) [(E)- 3-(4-methylphenyl) sulfonylprop-2-enenitrile], or the vehicle control, dimethylsulfoxide (DMSO), prior to treatment with 100 μM MnCl_2_. Pretreatment with Bay-11 broadly suppressed expression of inflammatory genes in mixed glia following exposure to 100 μM MnCl_2_, with significant suppression observed for *Nos2*, *Il6*, *Ccl5*, and *Ccl2* (Fig. 3a). Exposure to 100 μM MnCl_2_ in pure astrocytes resulted in significant increases in mRNA for most genes comparable to mixed glia (Fig. 3b), although *Nos2*, *Il6*, *Ccl5*, and *Ccl2* in pure astrocytes had a relatively larger fold-change in mRNA levels compared to mixed glia (Fig. 3b). Bay-11 decreased expression of *Nos2*, *Ccl5*, and *Ccl2* in pure astrocytes (Fig. 3b) but was less effective in decreasing expression of *C3*, *Il6*, *Il1β*, *Ccr2*, and *Tnfα* than in mixed glia.

### Pharmacologic inhibition of NF-κB in glia is neuroprotective

**Fig. 4:**
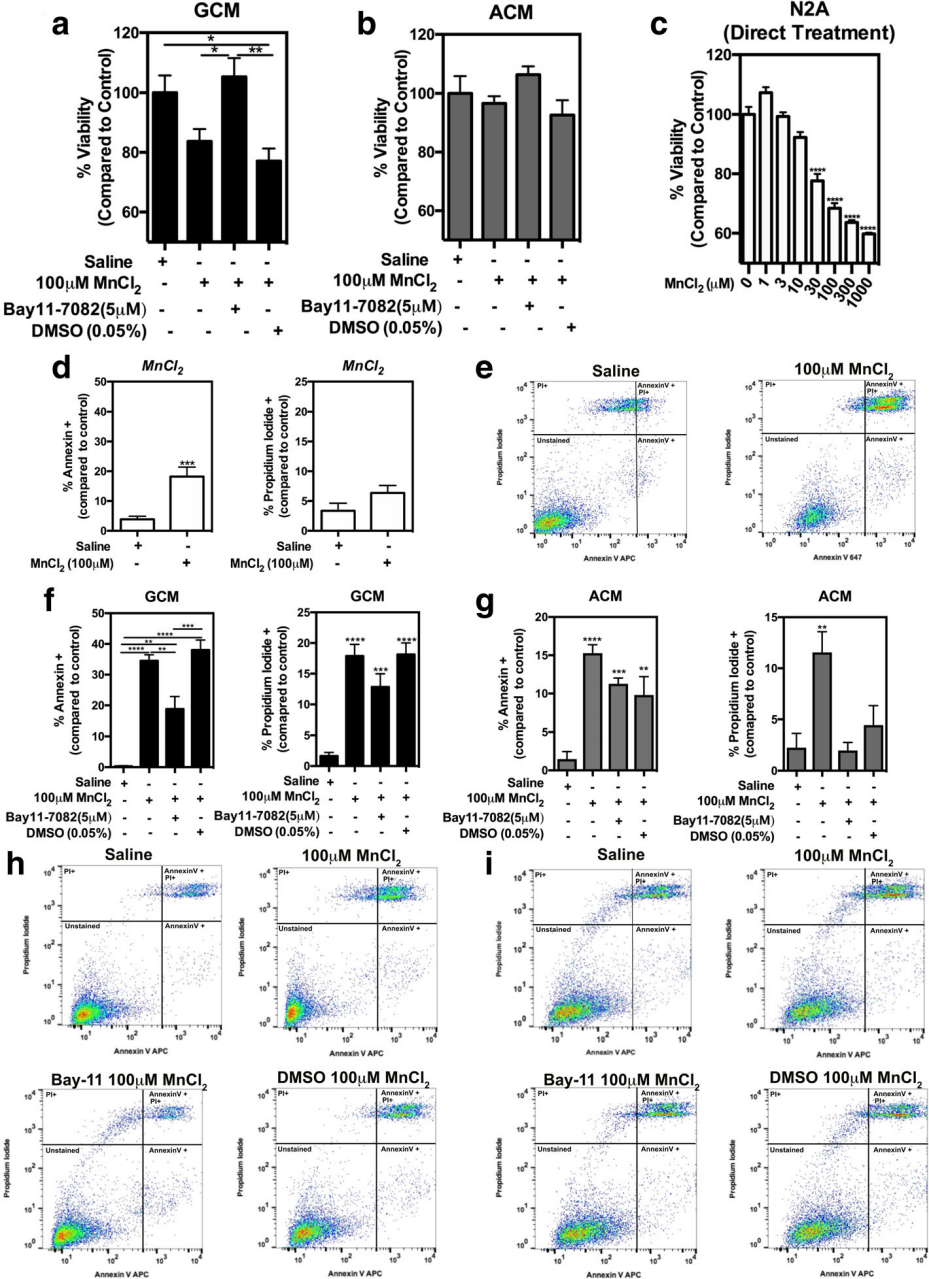
Pharmacologic inhibition of NF-κB in glia protects N2A neuronal cells from apoptosis induced by Mn-exposed glial conditioned media. Pharmacologic inhibition of NF-κB with Bay-11 in mixed glial cultures preserves N2A viability following treatment with 100 μM MnCl_2_ GCM (**a**) and ACM (**b**). Direct treatment of N2A cells with Mn causes a dose-dependent decrease in viability (**c**). Flow cytometry analysis of apoptotic N2A cells indicated that direct treatment with 100 μM MnCl_2_ (**d**, **e**) results in fewer Annexin V+ and PI+ cells compared to GCM (**f**, **h**) and ACM (**g**, **i**) and that pretreatment of glial cultures with Bay-11 prior to Mn exposure decreases the number of Annexin V+ and PI+ cells in both GCM- and ACM-treated N2A cells. One-way ANOVA analyses performed for experiments comparing three or more treatment groups and *T* test in those comparing two treatment groups. Data depicted as ± S.E.M. *P < 0.05; **P < 0.01; ***P < 0.001; ****P < 0.0001 (*n* ≥ 4 per treatment group; across ≥ 3 independent experiments)

To determine how glial-derived factors modulate neuronal viability following exposure to MnCl2, we examined the effect of Bay-11 treatment in glia on neuronal viability following incubation with GCM or ACM (Fig. 4). Pretreatment of mixed glia cultures with Bay-11 protected against Mn-induced loss of neuronal viability following incubation with GCM from Mn-treated glia, 105.2% viable compared to GCM-saline (Fig. 4a), whereas pre-treatment of mixed glia with vehicle control (DMSO) showed no protective effect and caused neuronal viability to decrease by ~ 23% compared to GCM-saline. There was a trend toward decreasing neuronal viability following incubation with ACM from Mn-treated astrocyte cultures that was reversed in cells treated with Bay-11, which had a relative viability of 106.3% compared to ACM-saline. DMSO co-treatment with ACM from Mn-treated astrocyte cultures showed no neuroprotective effect (Fig. 4b). To identify direct effects of Mn on neurons, N2A cells were incubated for 48 h with MnCl_2_ (1–1000 μM) and examined for viability. Treatment with increasing doses of MnCl_2_ resulted in loss of neuronal viability, with an LD_50_ value of 29.80 ± 1.5 μM (Fig. 4c). Analysis of N2A cells by flow cytometry following direct treatment with MnCl_2_ demonstrated increased numbers of apoptotic (+Annexin V) cells and a modest increase in dead (+Propidium Iodide (PI)) neurons (Fig. 4d, e). The magnitude of neuronal apoptosis following direct treatment with MnCl_2_ was markedly less than that induced by GCM or ACM. Flow cytometric analysis also demonstrated that Bay-11 treatment in mixed glia or pure astrocytes exposed to 100 μM MnCl_2_ was neuroprotective in both GCM (Fig. 4f, h) and ACM (Fig. 4g, i) treatment groups, respectively, based upon fluorescence intensity of Annexin V and PI.

### Gene deletion of IKK (NF-κB) in astrocytes decreases inflammatory gene expression

**Fig. 5:**
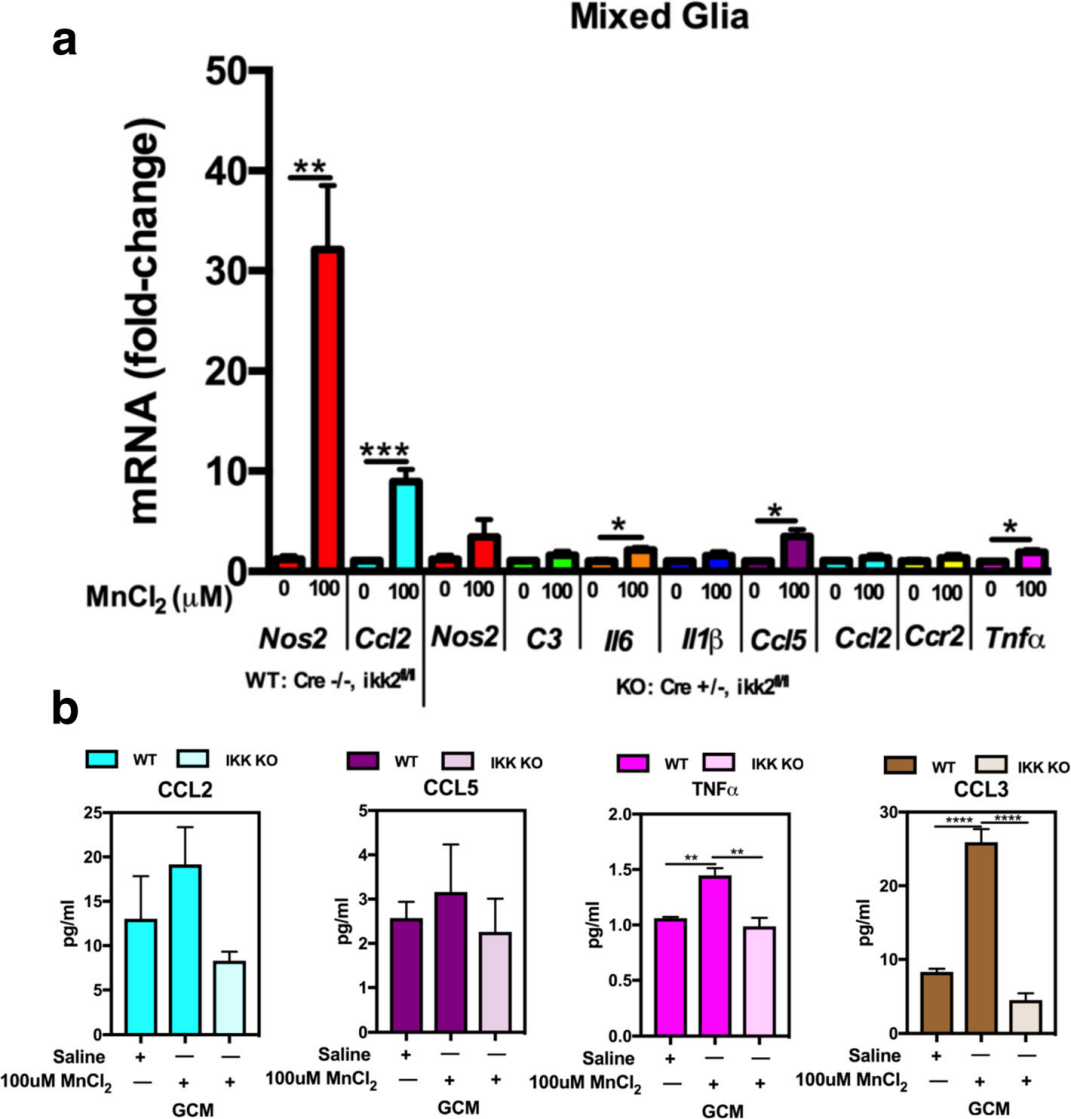
Gene deletion of IKK2 in astrocytes decreases inflammatory gene expression following exposure to 100 μM MnCl_2_ in mixed glial cultures. Astrocyte-specific IKK2 knockout decreases inflammatory mRNA (**a**) and protein (**b**) expression of inflammatory genes in mixed glial cultures compared to corresponding WT-treated groups. Two-way ANOVA analyses performed. Data depicted as ± S.E.M. *P < 0.05; **P < 0.01; ***P < 0.001; ****P < 0.0001 (*n* ≥ 4 per treatment group; across ≥ 3 independent experiments)

To determine the function of NF-κB in astrocyte-microglia inflammatory signaling, we treated mixed glial cultures containing IKK2 knockout (KO) astrocytes and wild-type microglia with 100 μM MnCl_2_ for 8 h and assessed mRNA expression of inflammatory genes (Fig. 5). These data demonstrate that astrocyte-specific knockout of IKK2 drastically decreases inflammatory gene expression for all genes including *Nos2*, *C3*, *Il6*, *Il1β*, *Ccl5*, *Ccl2*, *Ccr2*, and *Tnfα*, compared to mixed glia containing both littermate control wild-type astrocytes and microglia (Fig. 5a). Additionally, the resultant GCM from these experiments was examined by ELISA for protein levels of some of the genes targeted in mRNA studies, which also demonstrates that astrocyte-specific IKK knockout decreases protein levels of several NF-κB-regulated inflammatory genes (Fig. 5b).

### Genetic inhibition of astrocyte-specific NF-κB in glia is neuroprotective

**Fig. 6:**
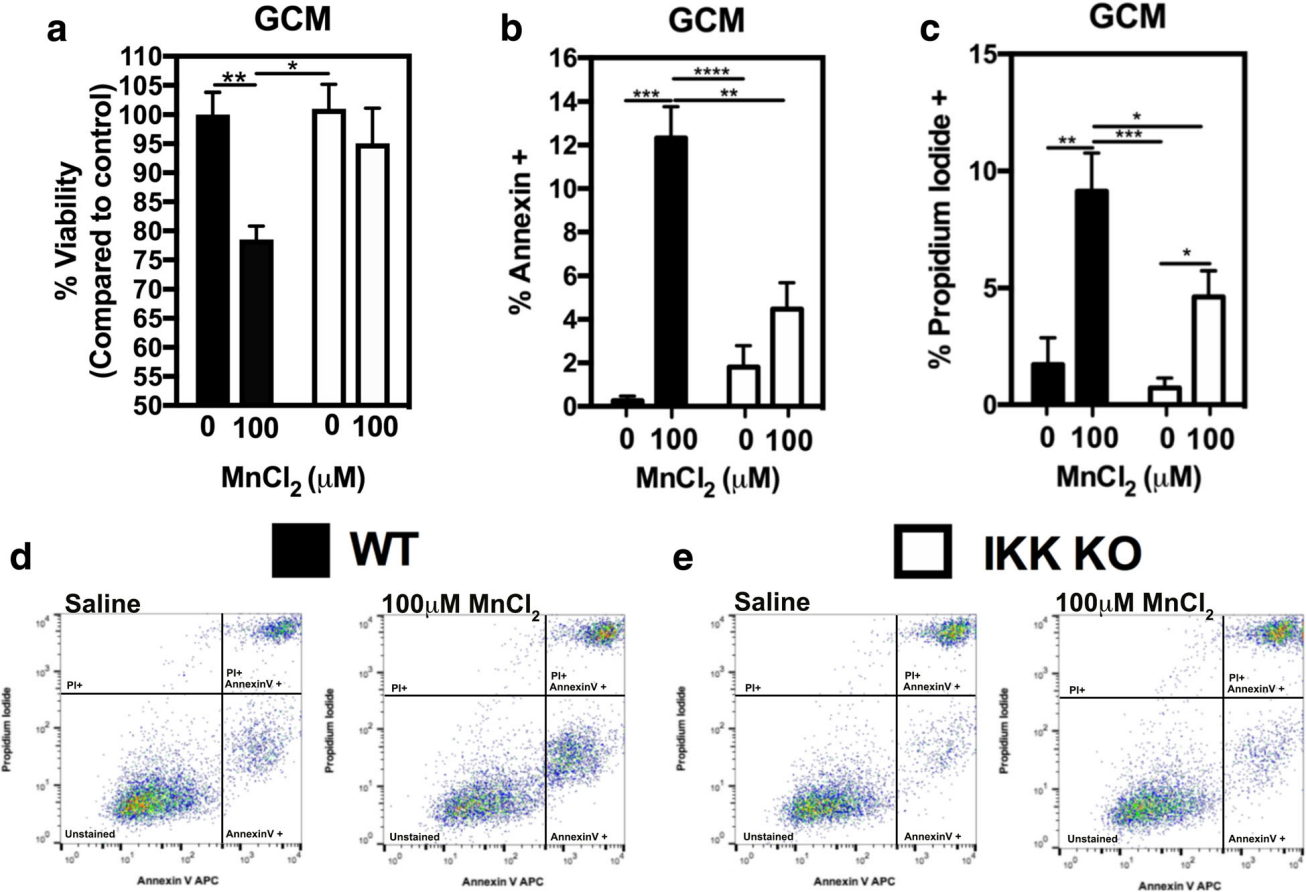
Loss of IKK2 expression in astrocytes reduces apoptosis in N2A cells following treatment with conditioned medium from mixed glial cultures. **a** GCM from mixed glial cultures containing WT astrocytes (black bars) treated with 100 μM MnCl_2_ decreases N2A viability compared to control (~ 25%), whereas neuronal viability is largely preserved (indicated by lack of statistically significant loss) in N2A cells treated with GCM from mixed glial cultures containing IKK2 KO astrocytes (white bars) compared to control. **b**, **c** Mn-GCM (WT, black bars) causes an increase in Annexin V+ and PI+ N2A cells, which is reduced in N2A cells treated with GCM from mixed glial cultures containing IKK2 KO astrocytes (white bars). Two-way ANOVA analyses performed. Data depicted as ± S.E.M. *P < 0.05; **P < 0.01; ***P < 0.001; ****P < 0.0001 (*n* ≥ 4 per treatment group; across ≥ 3 independent experiments)

To determine the role of IKK2/NF-κB in astrocytes in glial-mediated neuronal cell death during exposure to Mn, we assessed N2A cell viability after exposure to GCM from mixed glia containing IKK2 WT or KO astrocytes treated with saline or 100 μM MnCl_2_ (Fig. 6). These data indicate that GCM from mixed glia containing IKK KO astrocytes almost completely preserved N2A cell viability after exposure to 100 μM MnCl_2_, with only ~ 5% reduction in viability (Fig. 6a white bars) compared to exposure to GCM from mixed glia containing WT astrocytes, in which there was ~ 20% reduction in N2A cell viability (Fig. 6a black bars). Flow cytometric analysis of N2A cells exposed to IKK KO astrocyte GCM demonstrated a significant decrease in apoptotic (4.47% Annexin V+) (Fig. 6b white bars) and dead (4.62% PI+) (Fig. 6c white bars) N2A cells compared to wild-type GCM-100 μM MnCl_2_ (12.33% Annexin V+) and (9.14% PI+) (Fig. 6b, c black bars).

### Genetic inhibition of IKK (NF-κB) in astrocytes protects primary neurons from Mn-dependent inflammatory injury

**Fig. 7:**
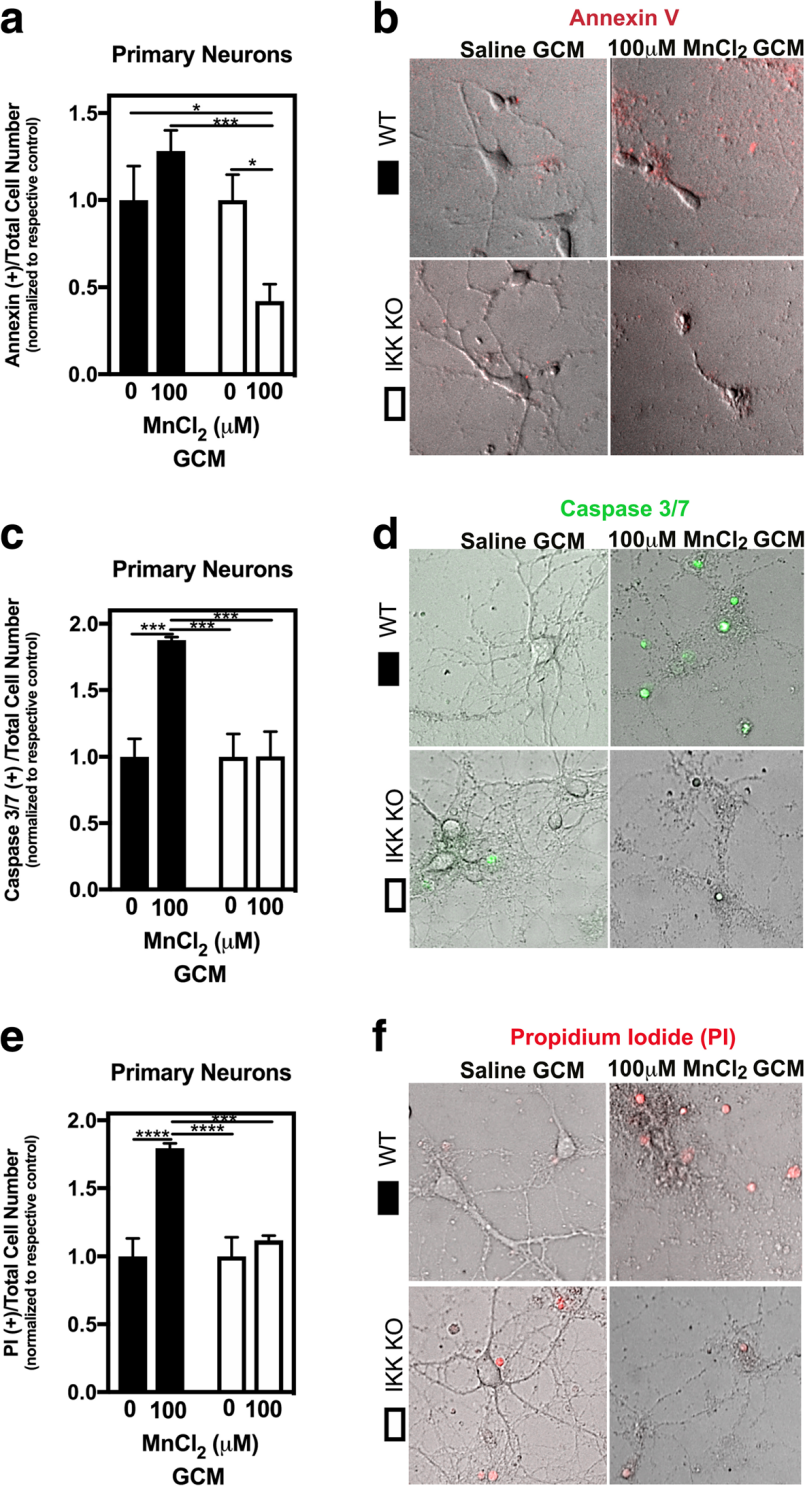
Mixed glial cultures containing IKK2 knockout astrocytes fail to induce apoptosis in primary cortical neurons following exposure to MnCl_2_. The number of apoptotic neurons is statistically decreased in response to Mn-treated IKK2 KO GCM compared to WT GCM for Annexin V+ cells (**a**, **b**) and caspase 3/7+ cells (**c**, **d**). Necrotic neurons (PI+; **e**, **f**) are similarly, statistically decreased in neurons treated with IKK2 KO GCM compared to WT GCM. Two-way ANOVA analyses performed. Data depicted as ± S.E.M. *P < 0.05; **P < 0.01; ***P < 0.001; ****P < 0.0001 (100–200 cells per group from four biological replicates across ≥ 3 independent experiments)

Additionally, we wanted to assess the effects of GCM from mixed glia containing IKK KO astrocytes and wild-type microglia on the viability of primary neurons. We therefore exposed primary neurons to GCM from Mn-treated mixed glia containing either WT astrocytes + WT microglia (WT) or IKK2 KO astrocytes + WT microglia (IKK KO)) for 48 h, similar to treatments done in N2A cells. Primary neurons exposed to WT GCM showed a significant ~ 5-fold increase in positive for Annexin V+ cells compared to those exposed to IKK2 KO GCM (Fig. 7a). Representative images demonstrating loss of neuronal morphology and increased Annexin V staining are depicted in cells exposed to WT GCM compared to IKK2 KO GCM and are depicted in Fig. 7b. Similarly, increases in caspase 3/7 activation (Fig. 7c, d) and in the number of PI+ cells (Fig. 7e, f) were detected in primary neurons exposed to WT but not IKK2 KO GCM.

### Knockdown of CCL2 in astrocytes reduces neuronal cell death from Mn-exposed mixed glial cultures

**Fig. 8:**
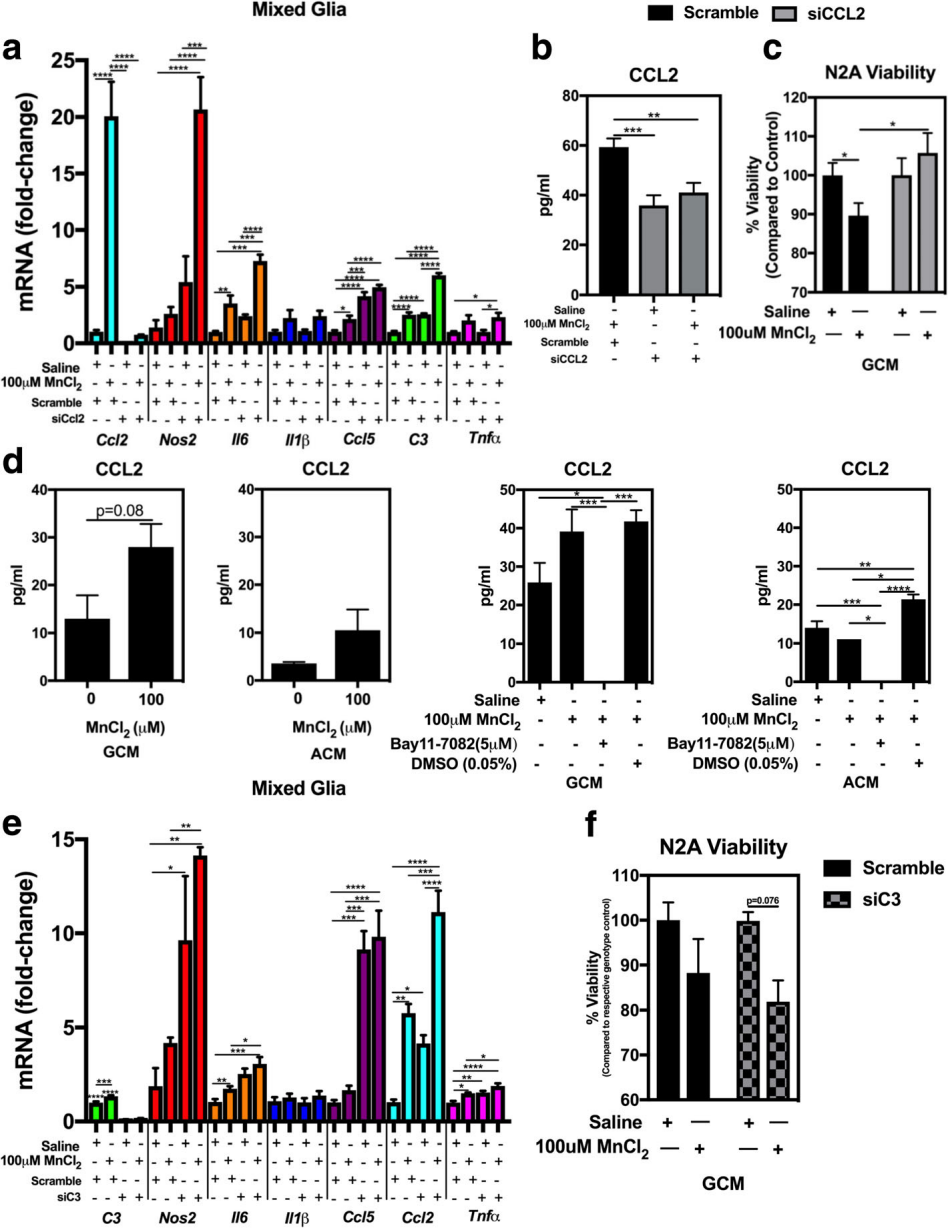
Selective knockdown of the astrocyte-specific chemokine, *Ccl2*, with siRNA in mixed glia does not inhibit Mn-induced inflammatory gene expression but prevents neuronal cell death. **a** *Ccl2* KD does not inhibit mRNA expression of inflammatory genes in Mn-exposed mixed glia compared to mixed glia treated with Mn in the presence of control siRNA. **b** CCL2 protein levels in GCM are reduced in *Ccl2* KD mixed glial cultures. **c** Viability of N2A cells is preserved following treatment with Mn-GCM from *Ccl2* KD mixed glia compared to those treated with control siRNA Mn-GCM. **d** (left two panels) CCL2 is released into medium in both mixed glia (GCM) and pure astrocytes (ACM) following treatment with MnCl_2_. **d** (right two panels) Pharmacologic inhibition of NF-κB with Bay-11 in both mixed glia and pure astrocyte cultures inhibits release of CCL2, whereas CCL2 levels are statistically increased compared to control in both Mn-GCM and Mn-ACM treated with vehicle control (DMSO). **e** *C3* KD does not inhibit mRNA expression of inflammatory genes in Mn-exposed mixed glia compared to mixed glia treated with Mn in the presence of control siRNA. **f** N2A viability is decreased in cells exposed to *C3* KD Mn-GCM compared to those treated with siRNA control Mn-GCM. One-way ANOVA analyses performed for experiments comparing three or more treatment groups and *t* test in those comparing two treatment groups. Data depicted as ± S.E.M. *P < 0.05; **P < 0.01; ***P < 0.001; ****P < 0.0001 (≥ 4 per treatment group; across ≥ 3 independent experiments)

Because expression of the astrocyte-specific chemokines, C3 and CCL2, is regulated by NF-κB and increased upon Mn exposure in mixed glia, we examined whether C3 or CCL2 gene expression in astrocytes modulates Mn-induced inflammatory responses in mixed glia (Fig. 8). We therefore used siRNA to knock down both C3 (~ 90% KD; Fig. 8e) and CCL2 (~ 95% KD; Fig. 8a, b) separately in mixed glial cultures prior to Mn exposure (Fig. 8a). Rather than causing a decrease in expression of other inflammatory genes, knockdown of C3 (Fig. 8e) and CCL2 (Fig. 8a) resulted in either no change or greater inflammatory gene induction compared to scramble control siRNA following Mn treatment. N2A viability assays revealed that GCM from CCL2 KD mixed glia resulted in preservation of neuronal viability following treatment with Mn-exposed GCM (Fig. 8c), whereas C3 knockdown had no effect on Mn-induced loss of neuronal cell viability (Fig. 8f). Additionally, ELISA assays demonstrated increased CCL2 in both mixed glial populations and in pure astrocyte populations following exposure to Mn (Fig 8d, left two panels), as well as levels of CCL2 that were below the limit of detection once mixed glia and pure astrocytes were exposed to Mn in the presence of Bay-11 (Fig. 8d, two right panels).

## Discussion

Mn-induced neurotoxicity in humans and in animal models is accompanied by reactive gliosis and inflammation that is damaging to surrounding neurons, although mechanisms regulating this neuroinflammatory phenotype remain poorly understood. Multiple studies have reported that microglia and astrocytes respond to Mn exposure with elevated levels of NF-κB-regulated inflammatory cytokines and other inflammatory mediators [11, 17, 25]. Furthermore, other studies have evaluated glial responses as a result of Mn-induced neuronal cell death [13]; however, research assessing the role that glial-glial and glial-neuronal communication plays in neurodegeneration in a model of manganism is lacking.

To identify glial-derived factors involved in neuronal injury as a result of Mn exposure, we first assessed Mn-induced inflammatory gene expression in three glial populations. We treated astrocytes and microglia, either together or separately, for 8 h with 0, 30, or 100 μM MnCl_2_. Overall, in mixed glial cell cultures, there was a dose-dependent increase in several NF-κB-regulated inflammatory genes including *Nos2*, *Il6*, and *Il1β* (Fig. 1a) that was much greater than in pure astrocytes (Fig. 1b) or pure microglia alone (Fig. 1c), suggesting that microglia-astrocyte communication amplifies the inflammatory response to Mn. Interestingly, the complement component *C3*, which is uniquely expressed by activated astrocytes in the CNS and correlates closely with a neurotoxic inflammatory phenotype (A1 astrocytes) [26], was more highly induced in mixed glia (~ 4-fold more in 100 μM compared to 0 μM) than in pure astrocytes (only ~ 2-fold increase), whereas pure microglia showed a dose-dependent decrease upon Mn-exposure, consistent with findings that show *C3* is not significantly expressed by microglia [26]. This demonstrates that *C3* is induced in astrocytes following Mn exposure and also that microglia potentiate expression of inflammatory gene expression in astrocytes, similar to in vivo studies reporting that release of the inflammatory cytokines C1q, IL1a, and TNF by microglia induces reactive astrocytosis in vivo [26]. Additionally, the chemokine, *Ccl2*, was induced following Mn exposure in mixed glia and in pure astrocytes but not in pure microglial cultures, consistent with studies demonstrating that *Ccl2* is astrocyte-derived and increases microglial activation and neuroinflammation [27, 28]. This suggests that cell-cell communication between microglia and astrocytes promotes an increase in astrocytic-specific inflammatory gene expression compared to astrocytes cultured in the absence of microglia, possibly through increased astrocyte expression of *Ccl2*. Other studies have also implicated CCL2-CCR2 signaling in astrocyte-mediated microglial activation in central nervous system (CNS) inflammation [27, 28], suggesting that the increase in both *Ccl2* and *Ccr2* in mixed glia is what contributes to an increased inflammatory gene response due to Mn treatment.

To determine whether glia release neurotoxic factors as a result of Mn exposure, we incubated N2A cells with conditioned media from cultures of mixed glia (GCM), astrocytes (ACM), or microglia (MCM) and found that 48 h of exposure to 100 μM Mn-GCM caused a greater decrease in neuronal viability compared to either ACM or MCM. Although purified astrocytes and microglia elicited some loss of neuronal viability in response to Mn, this was magnified with both cell types present, consistent with other studies demonstrating that Mn increases production in inflammatory cytokines and chemokines [5, 15, 17]. These data also suggest that cell-cell signaling between astrocytes and microglia amplifies the overall neuroinflammatory response to Mn, similar to recent studies from our lab demonstrating that microglia-derived inflammatory cytokines were greater in the presence of astrocytes than in pure microglial cultures [6]. Flow cytometric analysis also indicated that N2A cell death was enhanced by exposure to Mn-GCM, as shown by staining for Annexin V and Propidium Iodide for apoptotic and necrotic cells, respectively (Fig. 2c, d). This suggests that mixed populations of astrocytes and microglia produce more damaging levels of inflammatory mediators than either cell type alone, likely due to glial-glial communication that intensifies a reactive phenotype.

Because many inflammatory cytokines and chemokines associated with innate immune response in Mn-exposed glial cells are regulated by NF-κB [16, 25, 29], we tested the function of this signaling pathway in regulating astrocyte cross-communication with microglia. Treating purified astrocytes or mixed glia with the NF-κB/IKK2 inhibitor, Bay 11-7082, prior to treatment with 100 μM Mn resulted in significant reductions in glial activation, based on expression of inflammatory genes. The inflammatory genes upregulated (*Nos2*, *C3*, *Il6*, *Il1β*, *Ccl5*, *Ccl2*, *Ccr2*, and *Tnfα*) by Mn exposure were significantly decreased in both mixed glia and pure astrocytes, suggesting that Mn-induced inflammatory gene expression in mixed glia and astrocytes requires activation of NF-κB.

The functional effect of Bay-11 in Mn-treated glia was determined in neuronal viability studies using GCM or ACM derived from Bay-11-pretreated glial cells (Fig. 4). Bay-11 pretreatment was significantly neuroprotective (Fig. 4a) in GCM-treated N2A cells, whereas ACM showed a similar but less potent trend (Fig. 4b). Direct treatment of N2A cells with Mn demonstrated that the LD50 was 30 μM and that Mn also directly increased the number of apoptotic (Annexin V-positive) and necrotic (PI-positive) neuronal cells, although to a lesser extent than Mn-treated GCM or ACM (Fig. 4c–e). This finding supports that glial-derived factors contribute to N2A cell death. We previously reported that glial uptake of Mn is ~ 70%, leaving behind ~ 30% of Mn in the conditioned medium [6]; thus, uptake of 70% of 100 μM Mn treatment would result in ~ 30 μM in the resultant conditioned media, which caused greater cell death than direct treatment of N2A cells with 30 μM Mn. This finding suggests that N2A cell death from conditioned media exposure is not solely due to residual Mn in the media but is due in part to glial-released inflammatory factors, most likely regulated by NF-κB. This is further supported by the flow cytometric analysis of GCM and ACM from Bay-11 experiments in which there is increased neuroprotection as demonstrated by the significant decrease in apoptotic (Annexin V) and necrotic (PI) positive staining in N2A cells exposed to Bay-ll-pretreated GCM (Fig. 4f, h) or ACM (Fig. 4g, i). Taken together, these findings support the involvement of NF-κB activation in glial cells in the neurotoxicity of Mn by demonstrating that pharmacologic inhibition of NF-κB in glia is anti-inflammatory and neuroprotective.

To more precisely establish the involvement of NF-κB in Mn-induced glial inflammation and neuronal cell death, we isolated primary mixed glia from astrocyte-specific IKK knockout (KO) mice and exposed the mixed glia (containing IKK KO astrocytes and wild-type microglia) to 100 μM Mn for 8 h. We showed that there was a significant decrease in inflammatory gene expression based on levels of both mRNA and protein for multiple NF-κB-regulated inflammatory genes (Fig. 5), suggesting not only that NF-κB is directly involved in Mn-induced inflammatory gene expression, but also that genetically inhibiting NF-κB in astrocytes in a mixed glial culture mitigates overall expression of inflammatory genes in both astrocytes as well as microglia following exposure to Mn.

Exposing N2A cells to GCM from astrocyte-specific IKK2 knockout mixed glial cultures resulted in almost complete neuroprotection in GCM-treated N2A cells (Fig. 6a), with significantly fewer apoptotic (Annexin V) and necrotic (PI) positive staining (Fig. 6b, c). Additionally, incubation of primary cortical neurons with GCM from astrocyte-specific IKK2 knockout mixed glial cultures resulted in similar neuroprotection in live cell imaging experiments (Fig. 7), with decreased apoptotic (Annexin V), necrotic (PI), and active caspase 3/7-positive cells. This suggests that IKK2-dependent activation of NF-κB in astrocytes in response to Mn is critical for inflammatory injury to neurons, likely through the release of numerous neurotoxic inflammatory mediators that both directly injure neurons and amplify the inflammatory response of microglia.

To begin to identify astrocyte-derived factors that could result in amplification of glial inflammatory signaling and subsequent neuronal injury, we knocked down (KD) two NF-κB-mediated genes specific to astrocytes, *C3* and *Ccl2* [26, 27], and determined the effect on inflammatory gene expression and neuronal injury (Fig. 8). Knockdown of either *C3* or *Ccl2* did not diminish inflammatory gene expression in mixed glial cultures, indicating that these factors are not directly involved in glial cross-communication leading to amplification of inflammatory signaling. This is not surprising, given previous studies from our laboratory identifying TNF as a key regulator of glial reactivity in response to Mn [6]. However, TNF-dependent activation of NF-kB and subsequent production of C3 or CCL2 could still be directly toxic to associated neurons following exposure to Mn. To test this hypothesis, we incubated N2A cells with GCM from mixed glial cultures in which RNAi knocked down astrocytic expression of C3 or Ccl2. Treatment of N2A cells with GCM from C3 KD mixed glial cultures did not prevent Mn-dependent injury, whereas CCL2 KD GCM significantly decreased N2A cell death (Fig. 8c), demonstrating that CCL2 expression in astrocytes is necessary for glial-mediated neuronal cell death in response to Mn treatment. These data not only suggest that NF-κB activation in astrocytes is specifically involved in Mn-induced neuronal injury but also that astrocyte-derived CCL2 is a key contributor to neuronal cell death in a model of Mn neurotoxicity. The factor(s) regulated by CCL2 in microglia and astrocytes that cause direct injury to neurons following Mn exposure remain to be further elucidated.

## Conclusions

The mechanisms underlying neuronal death from exposure to Mn are not well understood but may be mediated by glial inflammation. Expression of neurotoxic inflammatory genes in glia is highly regulated through the NF-κB pathway, but the specific factors modulating neurotoxic glial-glial and glial-neuronal signaling by Mn are not well understood. The present data build on recently reported studies from our laboratory indicating that microglia amplify astrocyte activation during Mn exposure [6] and demonstrate that NF-κB in astrocytes stimulates the production of inflammatory cytokines and chemokines that cause neuronal cell death. Inhibition of NF-κB in astrocytes resulted in decreased inflammatory gene activation in mixed glia and increased neuroprotection, thus providing evidence that glial-glial and glial-neuronal communication through astrocyte-specific NF-κB is critical to mediating neurotoxic inflammatory signaling in response to Mn. Moreover, NF-κB-dependent expression of CCL2 in astrocytes appears to be critical to the inflammatory response to Mn exposure and to neuronal injury. Collectively, these data demonstrate that astrocyte-microglial signaling amplifies neuroinflammatory injury from Mn and suggests several inflammatory mediators regulated by NF-κB in astrocytes that likely influence the overall level of glial reactivity during exposure to Mn.

## Abbreviations

ACM: Astrocyte-conditioned media
CCL2: C-C motif chemokine ligand 2
CCL3: C-C motif chemokine ligand 3
CCL5: C-C motif chemokine ligand 5
CCR2: C-C chemokine receptor type 2
CD11b: C3: complement receptor
ELISA: Enzyme-linked immunosorbent assay
GCM: Glia-conditioned media
GFAP: Glial fibrillary acidic protein
GLAST: Glutamate transporter
IBA-1: Ionized calcium binding adaptor molecule 1
IL-6: Interleukin-6
IL-1β: Interleukin-1 beta
MCM: Microglia-conditioned media
Mn: Manganese
N2A: Neuro-2a
NF-κB: Nuclear factor kappa B
NOS2: Nitric oxide synthase 2
TNF: Tumor necrosis factor alpha
